# The influence of emotion dysregulation and perceived social support on the link between childhood emotional abuse and depressive symptoms in college students: a moderated mediation model

**DOI:** 10.3389/fpsyt.2025.1538390

**Published:** 2025-04-22

**Authors:** Huiyuan Huang, Haiqi Wu, Lin Luo, Bingqing Jiao, Yilin Wu, Guanyang Zou, Jiabao Lin, Wenqi Wang, Lijun Ma

**Affiliations:** ^1^ School of Public Health and Management, Guangzhou University of Chinese Medicine, Guangzhou, China; ^2^ State Key Laboratory of Cognitive Neuroscience and Learning, Beijing Normal University, Beijing, China

**Keywords:** childhood emotional abuse, perceived social support, emotion dysregulation, depressive symptoms, moderated mediation model

## Abstract

**Background:**

Childhood emotional abuse is strongly linked to an increased risk of depression. However, the pathways linking the two remain elusive. Our study sought to examine how emotion dysregulation and perceived social support influence the link between childhood emotional abuse and depressive symptoms among Chinese college students.

**Methods:**

This study involved 1728 Chinese college students aged 18-24. We utilized the Emotional Abuse (EA) subscale of the Childhood Trauma Questionnaire, Beck Depression Inventory (BDI), Difficulties in Emotion Regulation Scale (DERS) and Perceived Social Support Scale (PSSS) to assess the interrelationships among the study variables. A moderated mediation model was constructed to elucidate these relationships.

**Results:**

Our results indicated a positive correlation between EA, DERS, and BDI. Conversely, PSSS was negatively correlated with EA, BDI, and DERS. Notably, EA is linked to a heightened vulnerability to BDI, with DERS mediating this association. PSSS moderated both the direct path of EA on BDI and the association between DERS and BDI. Furthermore, gender difference was observed in the role of PSSS. PSSS moderated the link between EA and BDI was significant only in the male group and no longer significant under the condition of high PSSS.

**Conclusion:**

This study sheds light on the mediating effect of emotion dysregulation and the moderating effect of perceived social support in the connection between childhood emotional abuse and depressive symptoms. The adverse influence of childhood emotional abuse on subsequent depression may be mitigate through interventions focused on enhancing perceived social support and skills in regulating emotions in college students.

## 1 Introduction

Depression is a major health issue for college students. The epidemiological studies conducted among eight countries reported that 18.5% of college students experienced depressive disorder in the past twelve months, making it as the most prevalent psychiatric disorder (1). In China, the situation appears to be more pronounced, with over 20% of college students reporting suffering from depression (2, 3). The core symptoms of depression include persistent sense of sorrow and a reduced interest in activities, with additional symptoms such as lost appetite, insomnia or sleepiness, fatigue, inattention, and suicidal ideation (4). Depression among college students has an association with a range of negative consequence that can severely impact their well-being and academic life. Research has shown that depression correlates with an increased risk of sleep disturbance (5), internet addiction (6), impulse control disorders (7), and unhealthy behaviors such as excessive use of tobacco and alcohol (8, 9). These factors may diminish coping skills (10) and exacerbate academic stress, potentially impacting academic performance (11, 12). More seriously, depression is closely linked with the frequency of suicides (13, 14). Depression also imposes a considerable burden on college students. It is associated with strained family and social relationships, poor functioning, and immense economic expense (15). Given the clinical significance and pervasive occurrence of depression among college students, identifying its risk factors is crucial.

Childhood emotional abuse is a substantial etiologic contributor to depression. Childhood emotional abuse refers to a series of adverse life events that place children in an unsafe emotional environment that serves to satisfy the emotional needs of the abuser, including persuading children to commit inappropriate behavior and denigrating or destroying something they care about (16). Childhood emotional abuse can instill in children feelings of fear, guilt, or shame, which is the most enduring and damaging component of dysfunctional parent-child relationships (17). Compared with other subtypes in childhood maltreatment, childhood emotional abuse exerts a more robust and unequivocal influence on depression and leads to earlier onset, an attenuated therapeutic response, and heightened severity of depressive symptoms (18, 19). Childhood emotional abuse is a powerful indicator of depression among college students. For instance, a sample of 1317 college students from China demonstrated a notable connection between childhood emotional abuse and the prevalence of depression (20). One large sample study conducted on 30,374 Chinese college students identified that childhood emotional abuse was the most salient subtype of childhood abuse related to depression (21). However, not all college students with experiences of childhood emotional abuse will later suffer from depression, suggesting that the pathway that leads from childhood emotional abuse to depression may be more complex and dependent on other intervening variables. Therefore, it’s vital to clarify the processes that link childhood emotional abuse to depression. This understanding is important to the development of efficient strategies that alleviate depression among college students.

Childhood emotional abuse may severely impact the psychological and emotional functioning of vulnerable children, and its harm extends to adulthood (22). Emotion dysregulation is a crucial indicator in the association linking childhood emotional abuse to depression. Emotion regulation includes one or more processes by which individuals try to influence emotion in themselves or others to activate goals in short and/or long term (23, 24). Conversely, emotional dysregulation represents a maladaptive process characterized by the excessive intensity or protracted duration of emotional experiences or expressions (25). Thompson (26) proposed that the core of emotion dysregulation is the early relationships between children and parents, which affect children’s emotional development. Adverse early relationships can shape children’s perception of potential threats from adults and may lead to a heightened sensitivity to threatening stimuli and anger, which serve as a defensive mechanism (27, 28). Consequently, maltreated children may develop coping mechanisms that, while adaptive in their immediate environment, could lead to emotional maladaptation in the long run (26). It can also make them more vulnerable to negative emotions and develop a predisposition towards maladaptive strategies of emotional regulation, which may contribute to depression and other affective disorders (29). Prior research has indicated that emotion dysregulation may serve as a mediator to link childhood emotional abuse to depression across diverse groups. For instance, Zhang et al. (30) involving 7,041 adolescents suggested that maladaptive emotion regulation strategies serve as mediators to facilitate the association linking childhood emotional abuse to depression. Another cross-sectional study involving 3902 African-American participants with limited financial resources also confirmed that emotion dysregulation acted as a mediating role in linking childhood emotional abuse with depression (31). However, the existing cross-sectional study about the mediation effect of emotion dysregulation were either conducted with heterogeneous samples (e.g., adolescents or low-income adults) or focused on specific cognitive emotion regulation strategies (e.g., rumination and catastrophizing). The extent to which emotion dysregulation mediate the association linking childhood emotional abuse to depression among college students is less clear and requires further exploration, constituting a primary aim of this research. To date, only one study has indicated that emotion dysregulation serves as a mediator linking childhood emotional abuse to depression among college students. However, the sample is limited to 276 female students from the Netherlands (32). In summary, previous studies emphasize the imperative for broader and more comprehensive studies to ascertain the applicability of these results across the wider spectrum of college students.

Perceived social support is an internal resource that may contribute to buffering the enduring impacts of childhood emotional abuse, serving as a protective factor against depressive symptoms (33). Social support encompasses a range of dimensions, reflecting the various forms of emotional, practical, and advisory support individuals receive from diverse sources, including family members, partners, or peers (34). The concept is divided into actual social support and perceived social support. Perceived social support denotes the individual’s belief in the support they are likely to receive for their interpersonal relationships and social networks and the perceived efficacy and reliability of the expected assistance (35). Perceived social support represents individual differences to some extent, which better indicates psychological well-being than actual social support received (36, 37). That is, perceived social support assesses the anticipated help an individual can receive in times of need and is more reflective of the individual’s past experiences with support (38).Perceived social support is a mitigating indicator in reducing the likelihood of depression, which has been extensively validated in previous studies (39–41). A recent randomized controlled trial, which included 189 elderly participants, demonstrated that perceived social support can alleviate the severity of depression over 24 weeks (41). Furthermore, the detrimental effects of stress experienced in early life on psychological health may be lessened by perceived social support. The stress-buffering model posits that perceived social support lessens the repercussions of stressors (e.g., childhood emotional abuse) by moderating or averting a stress appraisal response. Sufficient perceived social support may enable individuals to reappraise stressful events cognitively, thus increasing their ability to handle emotional reactions and prevent potentially debilitating psychological consequences of stress (42). In line with the stress-buffering model, perceived social support could lessen the influence of childhood emotional abuse, thereby diminishing the propensity for depression. For example, a study involving 378 participants from the primary healthcare and obstetric-gynecological outpatient department demonstrated that perceived social support might alleviate the influence of childhood emotional abuse on depression only in women based on a hierarchical linear regression analysis (43). Additionally, a study that included 1396 Korean adults found that perceived quality of social relationships moderated the impact of childhood emotional abuse on depression among people with secondary education or lower (44). However, existing studies have predominantly concentrated on the moderating effects of other variables associated with perceived social support, including perceived quality of social relationships. To our knowledge, no existing studies have investigated whether perceived social support moderates the path of childhood emotional abuse on depression among college students.

Moreover, perceived social support could diminish the impact of emotion dysregulation on adverse psychological consequences. Xu et al. (45) have illustrated that perceived social support may reduce the influence of maladaptive emotion regulation strategies on suicidal ideation. However, the interplay of emotion dysregulation and perceived social support in relation to depression has rarely been explored in existing literature. The relational regulation theory asserts that perceived social support is inherently relational, reflecting individual preferences. It facilitates emotion regulation in general and effective social interactions (e.g., conversations), thereby reducing emotional disorders and promoting psychological well-being (46). Consequently, we propose that within social interaction contexts that are both effective and aligned with an individual’s cognitive and emotional inclinations, perceived social support is likely to ameliorate emotion dysregulation. This enhancement enables individuals to confront issues with a more positive disposition, thereby diminishing the likelihood of depression. Thus, our study proposes the hypothesis that perceived social support may exert a moderating influence of emotion dysregulation on depressive symptoms. Taken together, although the path between childhood emotional abuse and depression is well-established, the specific mechanisms through which emotion dysregulation and perceived social support influence this relationship among college students remain to be studied.

**Figure 1:**
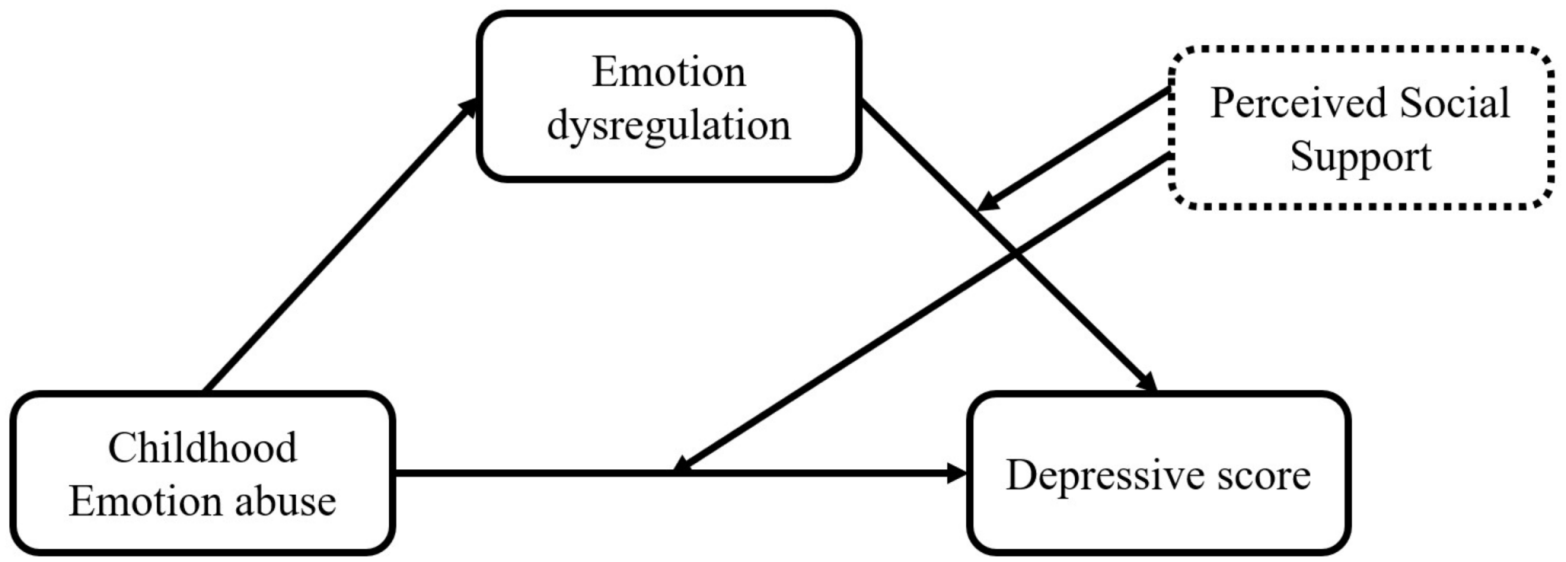
The conceptual framework of the moderated mediation model.

Our research attempted to explore the association between childhood emotional abuse and depression among Chinese college students, intending to delineate the mediating effect of emotion dysregulation as well as the moderating effect of perceived social support. Consequent to the synthesis of the existing literature, we hypothesized that: (1) emotion dysregulation mediates the association linking childhood emotional abuse to depressive scores. (2) Perceived social support moderates the direct path of childhood emotional abuse on depressive scores. (3) Perceived social support moderates the indirect path of emotion dysregulation on depressive scores. The conceptual framework of the proposed model can be found in **Figure 1**.

## 2 Methods

### 2.1 Participants

This study enrolled 2310 college students at Guangzhou University of Chinese Medicine. Participants were guided by trained investigators to complete standardized assessments. Exclusion criteria were as follows: (1) age less than 18, (2) unwillingness or inability to complete the questionnaire by the deadline, and (3) missing information/multiple fillings or inconsistencies in responses. This exclusion process ultimately comprised a sample of 1728 students with an effective recovery rate of 74.8%. Our final sample consists of 654 males and 1074 females, ranging from 18 to 24 years (mean age = 18.34 years old, standard deviation = 0.67). Participants provided written consent before taking part in the study. Ethical approval was granted by the Research Ethics Committee of the Second Affiliated Hospital of Guangzhou University of Chinese Medicine (IEC GL/07.0/01.1.).

### 2.2 Measures

#### 2.2.1 Childhood emotional abuse

Childhood emotional abuse was assessed using the Emotional abuse (EA) subscale of the Childhood Trauma Questionnaire-Short Form (CTQ-SF). The CTQ-SF is a 28-item self-report inventory designed to evaluate five subtypes of childhood abuse: emotional, physical, and sexual abuse, as well as emotional and physical neglect (47). The CTQ-SF utilized in this research was the Chinese rendition, which demonstrated strong internal reliability (48). The EA subscale comprises 5 questions, with respondents rating each item on a five-point Likert scale from “rarely” (score=1) to “frequently” (score=5). The EA score ranges from 0 to 25, with a score of 9 or above indicating the experience of childhood emotional abuse. An elevated total EA score is indicative of increased levels of experienced childhood emotional abuse (47). The scale has high reliability and validity among Chinese college students (21). The Cronbach’s α for the EA was 0.770.

#### 2.2.2 Emotion dysregulation

Emotion dysregulation was evaluated using the 36-item Difficulties in Emotion Regulation Scale (DERS). The scale comprises six subscales that evaluate various aspects of emotional experience and management: awareness of emotions, comprehension of emotions, acceptance of emotional responses, regulation of emotional urges, challenges in goal orientation, and difficulties in the effective application of emotional regulation strategies (49). Participants were required to answer how often the emotional experience as described by each item on a five-point Likert scale, with responses spanning from “rarely” (score=1) to “frequently” (score=5). The scale’s total score spans from 36 to 180, where an increased score corresponds to more emotion dysregulation (49). This scale has high reliability and validity in the Chinese samples (50). The Cronbach’s α for the DERS was 0.942.

#### 2.2.3 Perceived social support

The perceived social support scale (PSSS), comprising 12 items, assessed the levels of support perceived from diverse sources, including familial, amicable, and other important relationships (51). Participants are required to evaluate each item utilizing a seven-point Likert scale, from “completely disagree” (score=1) to “totally agree” (score=7). The aggregate score indicates the overall levels of PSSS, with scores spans from 12 to 84. An elevated score signifies a stronger perceived degree of social support. It has high reliability and validity in the Chinese population (52). The Cronbach’s α for the PSSS was 0.958.

#### 2.2.4 Depression

The Beck Depression Inventory (BDI) consists of 21 items designed to gauge the severity of depressive symptoms over the previous two weeks (53). Respondents score each question using a four-point Likert scale that spans from 0 to 3, where 0 indicates “I do not feel guilty” and 3 reflects “I felt guilty all the time.” The BDI score spans from 0 to 63, with higher scores is indicative of more severe depressive symptoms. Based on the criterion defined by Beck et al. (53), a score of 0-9 suggests no depression, 10-18 suggests mild depression, 19-29 suggests moderate depression, and 30-63 suggests severe depression. The BDI has demonstrated high reliability and validity among Chinese college student samples (54). The Cronbach’s α for the BDI was 0.890.

### 2.3 Data analysis

The analyses utilized SPSS 26.0 (IBM Corp., Armonk, NY, United States). First, a Harman’s single factor test was employed to test the reliability of the survey to avoid common method bias. Then, Univariate descriptive statistics applied to scrutinize the distribution of gender, age, EA, DERS, PSSS and BDI. Third, Pearson’s correlation analyses were executed to report the correlation of EA, DERS, PSSS and BDI. Fourth, multiple linear regression analyses coupled with a PROCESS macro program were deployed to assess the mediating and moderating influence of DERS and PSSS on the path between EA and BDI while controlling for gender and age. To avoid multicollinearity, all variables were mean-centered before constructing the interaction term when evaluating the moderating effect of PSSS. In addition, simple slope analyses were conducted to show significant interactions at one Standard Deviation (SD) below and one SD above the mean of PSSS. We also calculated 95% bootstrap confidence intervals (CI) based on 5000 bootstrap samplings. In addition, in order to investigate whether gender influences the moderated mediation model, we separated the participants into male and female groups, and preformed the same analyses in each group, with age as a covariate. To ensure model stability and the absence of multicollinearity, the tolerance (TOL) and variance inflation factor (VIF) were assessed and confirmed to be within acceptable thresholds (TOL > 0.1; VIF < 5).

## 3 Results

### 3.1 Common method bias control and inspection

The maximum variance extracted by a single factor was 23.6%, falling short of the threshold of 40% (55). Thus, this suggests that common method variance was not a significant concern in current research.

### 3.2 Demographics characteristics and preliminary statistics

The analyses encompassed 1728 college students, including 654 males (37.8%) and 1074 females (62.2%). The gender ratio was imbalanced but was consistent with prior research of medical universities (56–58). The mean age of our sample is 18.34 (SD=0.67, age of 18-24) years. The sample’s average EA score was 6.64 (SD=2.61). 285 participants (16.49%, score of 9-25) reported having experienced emotional abuse during childhood. The mean BDI score for our sample was 4.90 (SD = 6.33). 326 participants (18.87%) showed scores indicative of mild to severe depressive symptoms, which was in line with prior research among Chinese college students (2, 59). Specifically, 244 participants (14.12%) had mild depressive symptoms (score of 10-18), 72 participants (4.17%) had moderate depressive symptoms (score of 19-29), and 10 participants (0.58%) had severe depressive symptoms (score of 30-63) as classified by the criteria set by Beck, Steer (53). **Table 1** presents the summary of the descriptive statistics for all variables and the percentage of depressive symptoms.

**Table 1:** Descriptive statistics of the studied variables (*N*=1728).

Variable	Mean	SD	Range	n/N(%)
EA	6.64	2.61	5-25	
DERS	80.80	22.35	36-146	
PSSS	64.81	13.12	12-84	
BDI	4.90	6.33	0-45	
Mild depression	13.10	2.50	10-18	14.12%
Moderate depression	22.51	2.72	19-29	4.17%
Severe depression	34.90	4.46	30-63	0.58%

SD, standard deviation. EA, total score of the Emotional Abuse subscale of the Childhood Trauma Questionnaire-Short Form; DERS, total score of the Difficulties in Emotion Regulation Scale. PSSS, total score of the Perceived Social Support Scale; BDI, depressive score using the Beck Depression Inventory; n/N (%), percentage of different levels of depression.

### 3.3 The correlation among childhood emotional abuse, perceived social support, emotion dysregulation and depression

As expected, EA (*r* = 0.448, *p* < 0.001) and DERS (*r* = 0.534, *p* < 0.001) both showed a positive connection with BDI. In contrast, PSSS (*r* = -0.366, *p* < 0.001) had a negative relationship with BDI. Furthermore, EA demonstrated a significant positive link with DERS (*r* = 0. 375, *p* < 0.001) and showed a negative connection with PSSS (*r* = -0.334, *p* < 0.001). Additionally, DERS negatively correlated with PSSS (*r* = -0.460, *p* < 0.001). The bivariate correlation analyses results are delineated in **Table 2**.

**Table 2:** Intercorrelations between the studied variables (*N*=1728).

Variable	1	2	3
1. EA			
2. DERS	0.375***		
3. PSSS	-0.334***	-0.460***	
4. BDI	0.448***	0.534***	-0.366***

EA, total score of the Emotional Abuse subscale of the Childhood Trauma Questionnaire-Short Form; DERS, total score of the Difficulties in Emotion Regulation Scale; PSSS, total score of the Perceived Social Support Scale; BDI, depressive score using the Beck Depression Inventory. ***p<0.001

### 3.4 The mediating role of emotion dysregulation

The mediation effect of DERS in the connection link EA to BDI was detailed in **Table 3**. First, EA positively connected with DERS (*B* = 0.373, *p* < 0.001) in Model 1. Then, EA had a positive effect on BDI (*B* = 0.444, *p* < 0.001) in Model 2. Third, after controlling for EA, DERS maintained a positive effect on BDI (*B* = 0.425, *p* < 0.001) in Model 3. Despite EA retaining a positive connection with BDI (*B* = 0.286, *p <*0.001) in Model 3, its impact on BDI was attenuated after controlling for DERS relative to Model 2.

**Table 3:** Results of the path analyses (*N*=1728).

Predictor variable	DERS		BDI	
	Model 1	Model 2	Model 3	Model 4
Intercept	0.068	-0.783	-0.811	-0.806
Control variables				
Gender	-0.070	-0.071	-0.041	-0.056
Age (years old)	-0.002	0.044	0.045	0.042
Independent variable				
EA	0.373***	0.444***	0.286***	0.210***
Mediator				
DERS			0.425***	0.402***
Moderator				
PSSS		–		-0.094***
Interaction term				
EA × PSSS				-0.070***
DERS × PSSS				-0.080***
*R²*	0.142	0.203	0.358	0.381
*F*	94.682***	146.275***	240.253***	151.228***

Unstandardized regression coefficients are reported. EA, total score of the Emotional Abuse subscale of the Childhood Trauma Questionnaire-Short Form; DERS, total score of the Difficulties in Emotion Regulation Scale; PSSS, total score of the Perceived Social Support Scale; BDI, depressive score using the Beck Depression Inventory; ***p < 0.001.

In addition, we employed the SPSS Process Macro (60), Model 4, carry out a bootstrap method to reiterate the indirect path of DERS from EA to BDI, controlling for gender and age. The indirect effect of EA on BDI via the DERS (95% CI = [0.131, 0.189]) was significant. This result further demonstrated that emotion dysregulation mediated the pathway from childhood emotional abuse to depression among Chinese college students.

### 3.5 The moderating effect of perceived social support

**Figure 2:**
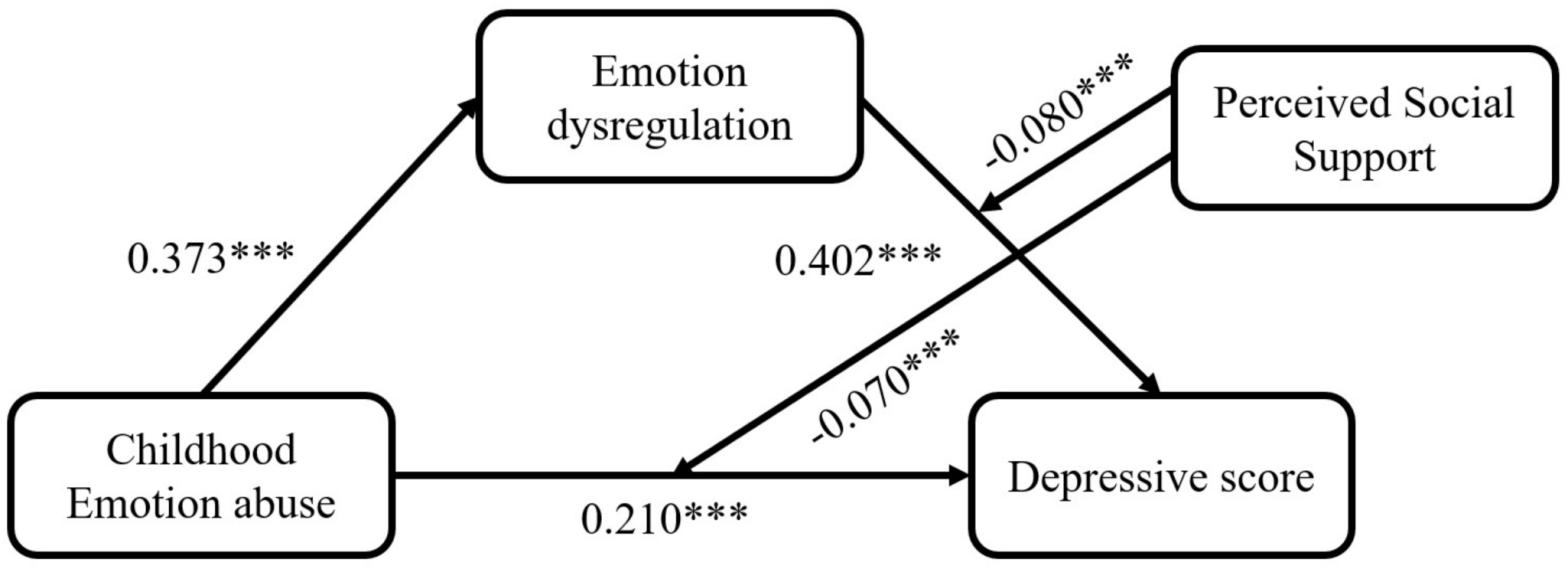
The moderated mediation model of the childhood emotional abuse, emotion dysregulation, perceived social support and depression. Path coefficients were shown in unstandardized regression coefficients; ***p<0.001.

**Figure 3:**
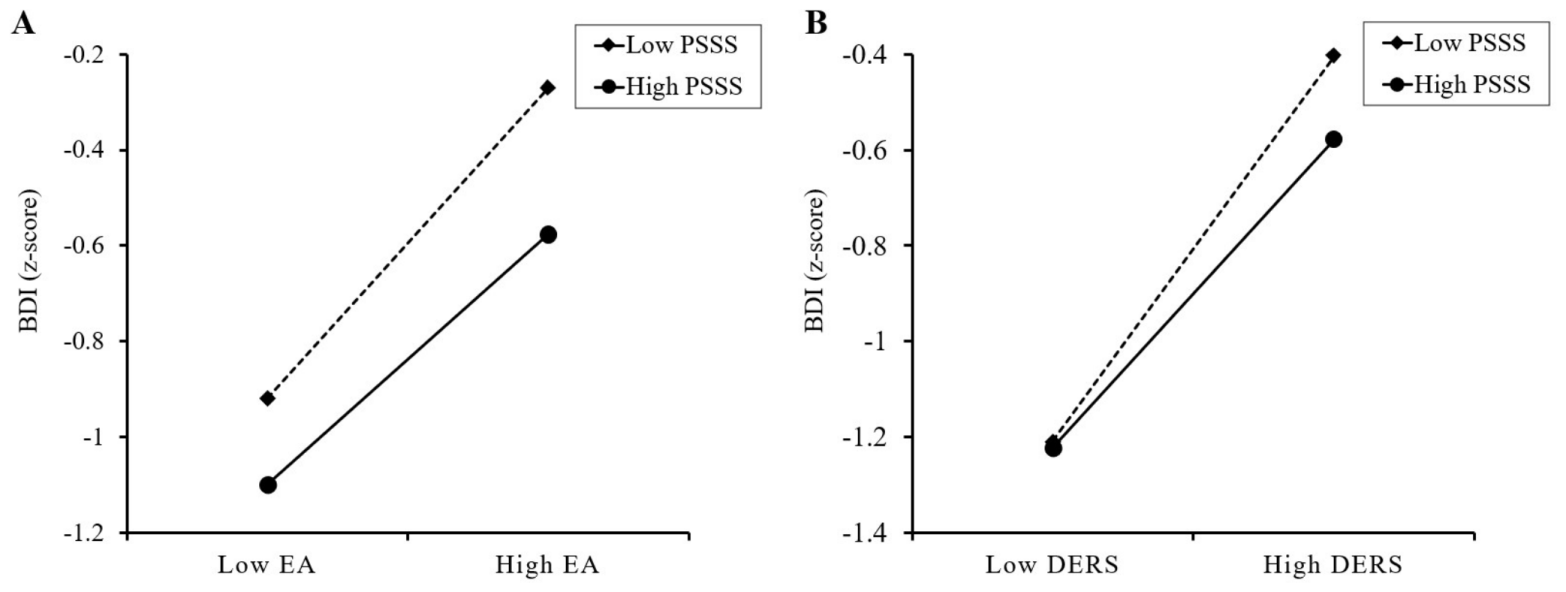
The results of simple slope analyses. **(A)** Perceived social support moderates the effect of childhood emotional abuse on depressive scores. **(B)** Perceived social support moderates the effect of difficulties in emotional regulation on depressive scores. EA, total score of the Emotional Abuse subscale of the Childhood Trauma Questionnaire-Short Form; DERS, total score of the Difficulties in Emotion Regulation Scale; PSSS, total score of the Perceived Social Support Scale; BDI, depressive score using the Beck Depression Inventory.

Our finding indicated that PSSS significantly moderated the direct influence of EA on BDI (*B* = -0.070, *p* < 0.001) and the link between DERS and BDI (*B* = -0.080, *p* < 0.001), as depicted in **Figure 2** and **Table 3**-Model 4. In addition, simple slope analyses were used to show the significant interaction at one SD below and one SD above the average of PSSS (See **Figure 3**). The results revealed that across varying levels of PSSS, elevated EA was correlated with higher BDI scores. In addition, regardless of the extent of PSSS, heightened DERS is linked to greater depressive symptoms. Specifically, the direct path between EA and BDI was significant with both low PSSS (*B*_simple_ = 0.280, *t* = 12.445, *p* < 0.001) and high PSSS (*B*_simple_ = 0.140, *t* = 3.785, *p* < 0.001). For the indirect path, the impact of DERS on BDI was also significant in both low PSSS (*B*_simple_ = 0.482, *t* = 15.828, *p* < 0.001) and high PSSS (*B*_simple_ = 0.322, *t* = 11.031, *p* < 0.001). However, the direct impact of EA on BDI was attenuated under high PSSS compared to low PSSS conditions (**Figure 3A**). The association between DERS and BDI was also weaker in the condition of high PSSS compared to low PSSS (**Figure 3B**).

In addition, we also used SPSS Process Macro (60), Model 15 to carry out a bootstrap method to reiterate the moderated mediation effect, taking gender and age as covariates. Our findings revealed that PSSS significantly moderated both the direct relationship of EA on BDI (*B* = -0.070, 95% CI = [-0.107, -0.032]) and the indirect path between EA and BDI through DERS (*B* = -0.080, 95% CI = [-0.119, -0.041]). The conditional direct and indirect effects between EA and BDI across varying degrees of PSSS were presented in **Table 4**, and pairwise contrasts of the conditional indirect effects between EA and BDI were presented in **Table 5**. Pairwise contrast in the conditional indirect effects between EA and BDI across varying degrees of PSSS indicated that the indirect influence for high PSSS (indirect effect = 0.120, 95%CI = [0.094, 0.149]) was significantly weaker than for those with low PSSS (indirect effect = 0.180, 95%CI = [0.142, 0.225]), as the zero was excluded from the 95% CI. This suggests that among individuals with greater PSSS, the connection link EA to BDI, mediated by DERS, is attenuated.

**Table 4:** Conditional direct and indirect effects of EA on BDI under different levels of PSSS.

	EA→BDI			EA→DERS→BDI		
	Effect	BootLLCL	BootULCI	Effect	BootLLCL	BootULCI
Effect 1 (M - 1SD)	0.280	0.236	0.324	0.180	0.142	0.225
Effect 2 (M)	0.210	0.163	0.257	0.150	0.123	0.181
Effect 3 (M +1SD)	0.140	0.068	0.214	0.120	0.094	0.149

EA, total score of the Emotional Abuse subscale of the Childhood Trauma Questionnaire-Short Form; PSSS, total score of the Perceived Social Support Scale; DERS, total score of the Difficulties in Emotion Regulation Scale; BDI, depressive score using the Beck Depression Inventory; M, mean; SD, standard deviation; BootLLCI, lower limit of 95% confidence interval; BootULCI, upper limit of 95% confidence interval.

**Table 5:** Pairwise contrasts between conditional indirect effects.

	PSSS	Effect	BootSE	BootLLCL	BootULCI
Pairwise contrasts between conditional indirect effects	Effect 2 - Effect 1	-0.030	0.009	-0.050	-0.013
	Effect 3 - Effect 1	-0.060	0.018	-0.098	-0.026
	Effect 3 - Effect 2	-0.030	0.009	-0.049	-0.013

PSSS, total score of the Perceived Social Support Scale; BootSE, standard error of bootstrap; BootLLCI, lower limit of 95% confidence interval; BootULCI, upper limit of 95% confidence interval.

### 3.6 Gender effects on the moderated mediation model

**Figure 4:**
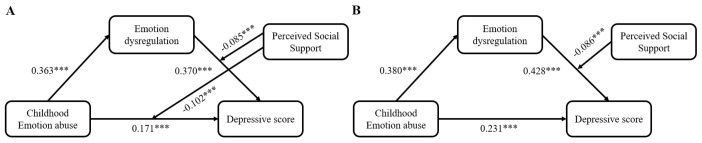
The moderated mediation model of the childhood emotional abuse, emotion dysregulation, perceived social support and depressive scores in the male group **(A)**, and in the female group **(B)**. Path coefficients were shown in unstandardized regression coefficients; ***p<0.001.

The detailed results of the gender effects on the moderated mediation model were displayed in the Supplementary Materials. In brief, the pattern of results from all samples was similar across both male and female groups. However, there are some gender differences in the studied variables. Demographics statistics showed that the scores of EA (*t* = -2.41, *p* = 0.016), DERS (*t* = -2.31, *p* = 0.016) and BDI (*t* = -2.35, *p* = 0.018) were significantly higher in female than in male. The moderating effect of PSSS on the direct path between EA and BDI pathway was significant only in the male group (*B* = -0.102, 95% CI = [-0.161, -0.044]), but not in the female group (*B* = -0.045, 95% CI = [-0.096, 0.007]) (See **Figure 4**). In addition, the direct effect of EA on BDI was significant in male with lower levels of PSSS, but was no longer significant in male with higher levels of PSSS.

## 4 Discussion

Given the harmfulness of childhood emotional abuse and depression among college students, our research developed a moderated mediation model to elucidate the mechanisms linking childhood emotional abuse to depression. Specifically, our findings can be summarized as follows: (1) Emotion dysregulation mediated the connection linking childhood emotional abuse to depressive symptoms; (2) Perceived social support moderated the direct relationship between childhood emotional abuse and depressive symptoms; (3) Perceived social support moderated the path of emotion dysregulation on depressive symptoms. Our results enhance the understanding of the processes that underpin depression in college students with emotional abuse in early life. Our results indicated that emotion dysregulation can make college students with childhood emotional abuse more vulnerable to negative emotions, consequently elevating their risk for depression. Furthermore, the results support the notion that perceived social support buffered against depression among college students. This protective effect is attributed to its capacity to mitigate not only the direct effects of childhood emotional abuse but also the influence of emotion dysregulation on depressive symptoms.

### 4.1 The mediating role of emotion dysregulation

Consistent with hypothesis 1, our study confirmed that emotion dysregulation had a mediation effect in the influence of childhood emotional abuse on depressive scores among college students. Aligning with existing literature (21, 29), our results indicated childhood emotional abuse was significantly positively connected with depressive scores (**Table 3**-Model 2). Many prior research had predominantly focused on the broader spectrum of childhood maltreatment or other specific subtypes in relation to depression (61–63), with less emphasis on the distinct impact of childhood emotional abuse. However, recent studies indicated that childhood emotional abuse may have a more significant impact on depression among college students (20). These findings supported the stress sensitization theory (64, 65), suggesting that childhood emotional abuse as a psychosocial stressor may leave vulnerabilities and increase sensitivity to the occurrence of subsequent emotional disorders, including depression. As expected, we identified that emotion dysregulation mediated the association linking childhood emotional abuse to depressive scores among Chinese college students (**Table 3**-Model 3). The mediation role of emotion dysregulation indicated that students who have experienced more childhood emotional abuse exhibit more severe of emotion dysregulation, which increases the likelihood of depression. Childhood is a pivotal period for the cultivation of emotional regulation ability, which is closely related to interactions with caregivers (66). In accordance with emotion socialization theory (67), long-term adverse emotional experiences, including punitive emotional responses from parents, are associated with children’s negative emotions and maladaptive coping. These experiences interfere with the development of effective emotional regulation strategies, potentially resulting in emotion dysregulation and internalizing disorders including major depression and anxiety disorder (66). Children who experience negative emotional responses, according to attachment theory, are prone to forming insecure attachments. Attachment theory further posits that children subjected to adverse emotional responses are prone to forming insecure attachments (68, 69), which may lead them to minimize or overregulate negative emotions in an attempt to activate the attachment (70). Therefore, negative interactions with caregivers (such as emotional abuse) can disrupt the normal development of emotion regulation, predisposing individuals to a higher risk of depression in adulthood. Our results may indicate that emotion dysregulation is the critical risk factor that predisposes individuals who have experienced childhood emotional abuse to be more vulnerable to developing depression. Inventions aimed at bolstering emotion regulation abilities, such as physical exercise interventions (jogging, yoga) (71, 72) or mindfulness-based practices (73), may assist college students in better recognizing and coping with negative emotions, thereby reducing the likelihood of developing depression.

### 4.2 The moderating role of perceived social support

Our study conducted further analysis to elucidate the moderating effect of perceived social support to understand how childhood emotional abuse has a direct and indirect association with the heightened risk of depression. Our results indicated that perceived social support moderated the direct path linking childhood emotional abuse to depression and weakened the positive connection linking emotion dysregulation to depression.

Consistent with hypothesis 2, perceived social support moderated the direct path link childhood emotional abuse to depressive scores (**Table 3**-model 4). Cheong et al. (74) found that robust perceived social support among individuals with childhood emotional abuse may benefit mental health. The stress-buffering model indicates that perceived social support can buffer the deleterious impact of stressors on individuals, thereby avoiding adverse outcomes such as depression (42). Perceived social support might serve as a buffer not only at the psychosocial level but also at the biological level, potentially influencing depression outcomes. Prior studies have revealed that early life stress, such as childhood emotional abuse, can trigger gene-environment interactions resulting in epigenetic alterations, including DNA methylation and dysregulated microRNA expression, which contribute to depression (75). However, social support, encompassing improvements in available resources, may mitigate the impact of adverse environments on such epigenetic modifications (76–78), thereby reducing the likelihood of depression. Recently, Lee, Stewart (79) demonstrated that the interactive effect of childhood emotional abuse and perceived social support can improve depression remission rate based on multinomial logistic regression tests. Our findings also suggest that perceived social support can inhibit and diminish the adverse influences of childhood emotional abuse on depression among college students. Specifically, Simple slope analyses revealed that perceived social support can lessen the positive association linking childhood emotional abuse to depressive symptoms regardless of its magnitude (**Figure 3A**). However, college students with higher perceived social support can diminish more influence of childhood emotional abuse on depression compared to those with lower perceived social support. Therefore, our results consist with previous studies (80, 81) indicated that enhancing the perceived social support among college students who have experienced childhood emotional abuse may attenuate their vulnerability to depressive symptoms. Notably, after the separate analyses of male and female groups, it was found that the moderating effect of perceived social support on the link between childhood emotional abuse and depression was significant only in the male group (**Figure 4**), which was also consistent with previous studies (77, 82), indicating that perceived social support may be more effective in alleviating early-life stress and reducing the likelihood of mental disorders in the male group. Moreover, under the condition of high level of perceived social support, the influence of childhood emotional abuse on depression was not significant in the male group (**Supplementary Table S5**).

Consistent with hypothesis 3, perceived social support attenuated the positive correlation linking emotion dysregulation to depressive symptoms, thereby weakening the mediation effect of emotion dysregulation (**Table 3**-Model 4). Specifically, the simple slope analyses revealed that college students with elevated levels of perceived social support were more capable of countering the impact of emotion dysregulation on depressive symptoms than those with lower levels of perceived social support (**Figure 3B**). Our results are in line with the Relational Regulation Theory, suggesting that perceived social support may attenuate the influence of emotion dysregulation on depression in regular social interaction. Moreover, our result was also in accordance with the Interpersonal Emotion Regulation Theory, which posits that social interactions can facilitate the employ of adaptive emotion regulation strategies (e.g., distraction), leading to a reduced attentional deployment towards depressive stimuli (36). Individuals with robust perceived social support are conducive to interact with significant others actively. Within the process of positive social interaction, individuals are likely to shift their focus away from negative emotions and towards positive stimuli, consequently mitigating depressive affect. A recent study has also indicated that good interpersonal interactions can blunt the impact of maladaptive emotion-regulation strategies, thereby reducing the propensity for the onset of depressive symptoms (83). In addition, previous study confirmed that perceived social support from familial, amicable, and intimate relationships exerts a protective effect irrespective of the intensity of childhood abuse endured (33). Previous research has indicated that individuals who have experienced severe childhood emotional abuse may perceive lower levels of social support (84). Therefore, it is crucial to place greater emphasis on the social support from family members, friends, and significant others. The implications of our study imply that interventions aimed at boosting perceived social support from familial, amicable, and intimate relationships should be prioritized to lessen the adverse effects of emotion dysregulation on depression for college students with childhood emotional abuse.

## 5 Limitation

Several limitations should be acknowledged. First, our study was conducted in a general population of Chinese college students, caution is urged in terms of the findings’ generalizability to other samples (e.g., clinically depressed patients) and other cultural contexts. Subsequent studies should extend to more diverse groups to validate the observations. Second, our sample exhibited a gender skew, with a ratio of male to female of 1:1.64, mirroring the gender distribution in prior research in Chinese medical universities (57, 85). Despite this, we have statistically controlled for gender effects to mitigate the potential bias introduced by this imbalance. In addition, the role of perceived social support in the association between childhood emotional abuse and depression is different between males and females, future studies can further explore potential reasons for such gender difference. Third, the reliance on retrospective self-report questionnaires for data collection could be influenced by the participants’ recall accuracy and current emotional state, potentially introducing reporting bias. Future studies could employ a variety of methodologies (e.g., records from child protective services, observational or physiological assessments of emotion regulation, and interviews for depression evaluation) to corroborate these results. Fourth, the cross-sectional design precludes definitive conclusions about causality between the variables examined. A further longitudinal study to explore why and how childhood emotional abuse may influence depression through emotion dysregulation and perceived social support is necessary. Fifth, this study focused exclusively on the severity of childhood emotional abuse, without collecting detailed information on factors such as the time of exposure, duration, or other specific characteristics of childhood emotional abuse exposure. Additionally, demographic variables such as socioeconomic status (SES) were not comprehensively assessed. Future research would benefit from collecting more detailed data on childhood emotional abuse exposure and sociodemographic covariates to account for potential confounding effects, and to draw more definitive conclusions regarding the pathways through which childhood emotional abuse contributes to depression. Furthermore, our research focuses on individuals’ subjective experiences of perceived social support. Both perceived social support and actual social support were assessed through participants’ retrospective self-reports, whereas actual social support assesses more specific and recent events of support, rather than reflecting a general pattern of social interaction with others (86). Moreover, the concept of perceived social support aligns more closely with the construct of “social support” as defined in the stress-buffering model. Previous studies suggested that higher levels of perceived social support is associated with reduced depressive symptoms, and may be more effective than actual social support in alleviating the influence of stress to adverse outcome (87, 88). Future studies should further verify the role of actual social support in the association between childhood emotional abuse and depression.

## 6 Conclusion

This study constructed a moderated mediation model and supported the potential mediating effect of emotion dysregulation and the moderating effect of perceived social support in the association linking childhood emotional abuse to depressive symptoms. Our results contribute to a more nuanced comprehension of the underlying external and internal factors that affect the association linking childhood emotional abuse to depression. Prevention and intervention strategies aim at fostering adaptive emotional regulation strategies and bolstering perceived social support may prove efficacious in improving mental health and reducing depression in college students with emotional abuse in early life.

## Data Availability

The original contributions presented in the study are included in the article/**Supplementary Material**, further inquiries can be directed to the corresponding author/s.

## References

[1] Auerbach RP Mortier P Bruffaerts R Alonso J Benjet C Cuijpers P. Who world mental health surveys international college student project: prevalence and distribution of mental disorders. J Abnormal Psychol. (2018) 127:623–38. doi: 10.1037/abn0000362 PMC6193834 30211576

[2] Fu H Pan M Lai M. Sources of Negative Emotions and Tactics of Self-Emotion Regulation among College Students during Covid-19 School Closure in China. Front Public Health. (2024) 12:1265350. doi: 10.3389/fpubh.2024.1265350 38572013 PMC10987727

[3] Zhang Y Tao S Qu Y Mou X Gan H Zhou P. The Correlation between Lifestyle Health Behaviors, Coping Style, and Mental Health during the Covid-19 Pandemic among College Students: Two Rounds of a Web-Based Study. Front Public Health. (2023) 10:1031560. doi: 10.3389/fpubh.2022.1031560 36711327 PMC9878348

[4] APA. Diagnostic and Statistical Manual of Mental Disorders. Fifth Edition. Washington, DC: American Psychiatric Association (2013).

[5] Nyer M Farabaugh A Fehling K Soskin D Holt D Papakostas GI. Relationship between sleep disturbance and depression, anxiety, and functioning in college students. Depression Anxiety. (2013) 30:873–80. doi: 10.1002/da.22064 PMC3791314 23681944

[6] Peterka-Bonetta J Sindermann C Sha P Zhou M Montag C. The relationship between internet use disorder, depression and burnout among Chinese and German College students. Addictive Behav. (2019) 89:188–99. doi: 10.1016/j.addbeh.2018.08.011 30321691

[7] Leppink EW Lust K Grant JE. Depression in university students: associations with impulse control disorders. Int J Psychiatry Clin Pract. (2016) 20:146–50. doi: 10.1080/13651501.2016.1197272 27314569

[8] Morrell HER Cohen LM McChargue DE. Depression vulnerability predicts cigarette smoking among college students: gender and negative reinforcement expectancies as contributing factors. Addictive Behav. (2010) 35:607–11. doi: 10.1016/j.addbeh.2010.02.011 PMC2838988 20181432

[9] Collins J-L Thompson K Sherry SB Glowacka M Stewart SH. Drinking to cope with depression mediates the relationship between social avoidance and alcohol problems: A 3-wave, 18-month longitudinal study. Addictive Behav. (2018) 76:182–7. doi: 10.1016/j.addbeh.2017.08.020 28846938

[10] Zong J-G Cao X-Y Cao Y Shi Y-F Wang Y-N Yan C. Coping flexibility in college students with depressive symptoms. Health Qual Life Outcomes. (2010) 8:1–6. doi: 10.1186/1477-7525-8-66 PMC2911409 20626865

[11] Alhamed AA. The Link among Academic Stress, Sleep Disturbances, Depressive Symptoms, Academic Performance, and the Moderating Role of Resourcefulness in Health Professions Students during Covid-19 Pandemic. J Prof Nurs. (2023) 46:83–91. doi: 10.1016/j.profnurs.2023.02.010 37188428 PMC10020862

[12] Letina S Quinn DM Canevello A Crocker JK. Understanding the role of depressive symptoms in academic outcomes: A longitudinal study of college roommates. PloS One. (2023) 18. doi: 10.1371/journal.pone.0286709 PMC10241356 37276215

[13] Rotenstein LS Ramos MA Torre M Segal JB Peluso MJ Guille C. Prevalence of depression, depressive symptoms, and suicidal ideation among medical students: A systematic review and meta-analysis. Jama. (2016) 316:2214–36. doi: 10.1001/jama.2016.17324 PMC5613659 27923088

[14] Pan YJ Juang KD Lu SR Chen SP Wang YF Fuh JL. Longitudinal risk factors for suicidal thoughts in depressed and non-depressed young adolescents. Aust N Z J Psychiatry. (2017) 51:930–7. doi: 10.1177/0004867417717795 28701051

[15] Karyotaki E Klein AM Ciharova M Bolinski F Krijnen L de Koning L. Guided internet-based transdiagnostic individually tailored cognitive behavioral therapy for symptoms of depression and/or anxiety in college students: A randomized controlled trial. Behav Res Ther. (2022) 150. doi: 10.1016/j.brat.2021.104028 35066365

[16] Teicher MH Samson JA Anderson CM Ohashi K. The effects of childhood maltreatment on brain structure, function and connectivity. Nat Rev Neurosci. (2016) 17:652–66. doi: 10.1038/nrn.2016.111 27640984

[17] Rees CA. Understanding emotional abuse. Arch Dis Child. (2010) 95:59–67. doi: 10.1136/adc.2008.143156 20040686

[18] Teicher MH Gordon JB Nemeroff CB. Recognizing the importance of childhood maltreatment as a critical factor in psychiatric diagnoses, treatment, research, prevention, and education. Mol Psychiatry. (2021) 27:1331–8. doi: 10.1038/s41380-021-01367-9 PMC8567985 34737457

[19] Nelson J Klumparendt A Doebler P Ehring T. Childhood maltreatment and characteristics of adult depression: meta-analysis. Br J Psychiatry. (2018) 210:96–104. doi: 10.1192/bjp.bp.115.180752 27908895

[20] Wang W Wang X Duan G. Non-suicidal self-injury and suicidal ideation among Chinese college students of childhood emotional abuse: associations with rumination, experiential avoidance, and depression. Front Psychiatry. (2023) 14:1232884. doi: 10.3389/fpsyt.2023.1232884 37588028 PMC10427149

[21] Li Q Guo L Zhang S Wang W Li W Chen X. The relationship between childhood emotional abuse and depressive symptoms among Chinese college students: the multiple mediating effects of emotional and behavioral problems. J Affect Disord. (2021) 288:129–35. doi: 10.1016/j.jad.2021.03.074 33878646

[22] Lippard ETC Nemeroff CB. The devastating clinical consequences of child abuse and neglect: increased disease vulnerability and poor treatment response in mood disorders. Am J Psychiatry. (2020) 177:20–36. doi: 10.1176/appi.ajp.2019.19010020 31537091 PMC6939135

[23] McRae K Gross JJ. Emotion regulation. Emotion. (2020) 20:1–9. doi: 10.1037/emo0000703 31961170

[24] Sheppes G Suri G Gross JJ. Emotion regulation and psychopathology. Annu Rev Clin Psychol. (2015) 11:379–405. doi: 10.1146/annurev-clinpsy-032814-112739 25581242

[25] Vogel AC Brotman MA Roy AK Perlman SB. Review: defining positive emotion dysregulation: integrating temperamental and clinical perspectives. J Am Acad Child Adolesc Psychiatry. (2023) 62:297–305. doi: 10.1016/j.jaac.2022.06.019 36007814 PMC11323061

[26] Thompson RA. Emotion dysregulation: A theme in search of definition. Dev Psychopathol. (2019) 31:805–15. doi: 10.1017/s0954579419000282 31030684

[27] Fani N Bradley-Davino B Ressler KJ McClure-Tone EB. Attention bias in adult survivors of childhood maltreatment with and without posttraumatic stress disorder. Cogn Ther Res. (2011) 35:57–67. doi: 10.1007/s10608-010-9294-2

[28] Russo M Mahon K Shanahan M Solon C Ramjas E Turpin J. The association between childhood trauma and facial emotion recognition in adults with bipolar disorder. Psychiatry Res. (2015) 229:771–6. doi: 10.1016/j.psychres.2015.08.004 PMC4603568 26272021

[29] Zhou X Zhen R. How do physical and emotional abuse affect depression and problematic behaviors in adolescents? The roles of emotional regulation and anger. Child Abuse Negl. (2022) 129:105641. doi: 10.1016/j.chiabu.2022.105641 35487046

[30] Zhang Y Xu W McDonnell D Wang J-L. The relationship between childhood maltreatment subtypes and adolescent internalizing problems: the mediating role of maladaptive cognitive emotion regulation strategies. Child Abuse Negl. (2024) 152. doi: 10.1016/j.chiabu.2024.106796 38631188

[31] Crow T Cross D Powers A Bradley B. Emotion dysregulation as a mediator between childhood emotional abuse and current depression in a low-income african-american sample. Child Abuse Negl. (2014) 38:1590–8. doi: 10.1016/j.chiabu.2014.05.015 PMC4254147 25035171

[32] Christ C de Waal MM Dekker JJM van Kuijk I van Schaik DJF Kikkert MJ. Linking childhood emotional abuse and depressive symptoms: the role of emotion dysregulation and interpersonal problems. PloS One. (2019) 14:e0211882. doi: 10.1371/journal.pone.0211882 30763360 PMC6375578

[33] Evans SE Steel AL DiLillo D. Child maltreatment severity and adult trauma symptoms: does perceived social support play a buffering role? Child Abuse Negl. (2013) 37:934–43. doi: 10.1016/j.chiabu.2013.03.005 PMC3758446 23623620

[34] Gariepy G Honkaniemi H Quesnel-Vallee A. Social support and protection from depression: systematic review of current findings in western countries. Br J Psychiatry. (2016) 209:284–93. doi: 10.1192/bjp.bp.115.169094 27445355

[35] Schulz P Beblo T Ribbert H Kater L Spannhorst S Driessen M. How is childhood emotional abuse related to major depression in adulthood? The role of personality and emotion acceptance. Child Abuse Negl. (2017) 72:98–109. doi: 10.1016/j.chiabu.2017.07.022 28787645

[36] Marroquin B. Interpersonal emotion regulation as a mechanism of social support in depression. Clin Psychol Rev. (2011) 31:1276–90. doi: 10.1016/j.cpr.2011.09.005 21983267

[37] McDowell TL Serovich JM. The effect of perceived and actual social support on the mental health of hiv-positive persons. AIDS Care. (2007) 19:1223–9. doi: 10.1080/09540120701402830 PMC2151198 18071966

[38] Ibarra-Rovillard MS Kuiper NA. Social support and social negativity findings in depression: perceived responsiveness to basic psychological needs. Clin Psychol Rev. (2011) 31:342–52. doi: 10.1016/j.cpr.2011.01.005 21382539

[39] Dour HJ Wiley JF Roy-Byrne P Stein MB Sullivan G Sherbourne CD. Perceived social support mediates anxiety and depressive symptom changes following primary care intervention. Depression Anxiety. (2014) 31:436–42. doi: 10.1002/da.22216 PMC4136523 24338947

[40] Fitzgerald M Gallus K. Emotional support as a mechanism linking childhood maltreatment and adult’s depressive and social anxiety symptoms. Child Abuse Negl. (2020) 108. doi: 10.1016/j.chiabu.2020.104645 32799014

[41] Woods A Solomonov N Liles B Guillod A Kales HC Sirey JA. Perceived social support and interpersonal functioning as predictors of treatment response among depressed older adults. Am J Geriatric Psychiatry. (2021) 29:843–52. doi: 10.1016/j.jagp.2020.12.021 PMC8255325 33419660

[42] Cohen S Wills TA. Stress, social support, and the buffering hypothesis. psychol Bull. (1985) 98:310. doi: 10.1037/0033-2909.98.2.310 3901065

[43] Powers A Ressler KJ Bradley RG. The protective role of friendship on the effects of childhood abuse and depression. Depression Anxiety. (2009) 26:46–53. doi: 10.1002/da.20534 18972449 PMC2629811

[44] Jung JH Soo SHJ. Childhood emotional abuse and adult mental health at the intersection of social relationship and education. Int J Soc Psychiatry. (2023) 69:1335–44. doi: 10.1177/00207640231161295 36967579

[45] Xu W Shen X McDonnell D Wang J. Childhood maltreatment and suicidal ideation among Chinese adolescents: moderated mediation effect of perceived social support and maladaptive cognitive emotion regulation strategies. Child Abuse Negl. (2024) 151:106732. doi: 10.1016/j.chiabu.2024.106732 38503245

[46] Lakey B Orehek E. Relational regulation theory: A new approach to explain the link between perceived social support and mental health. psychol Rev. (2011) 118:482–95. doi: 10.1037/a0023477 21534704

[47] Bernstein DP Stein JA Newcomb MD Walker E Pogge D Ahluvalia T. Development and validation of a brief screening version of the childhood trauma questionnaire. Child Abuse Negl. (2003) 27:169–90. doi: 10.1016/s0145-2134(02)00541-0 12615092

[48] Zhao X Zhang Y Li L Zhou Y Li H Yang S. Reliability and validity of the chinese version of childhood trauma questionnaire. Chin J Tissue Eng Res. (2005) 20:105–7. doi: 10.3321/j.issn:1673-8225.2005.20.052

[49] Gratz KL Roemer L. Multidimensional assessment of emotion regulation and dysregulation: development, factor structure, and initial validation of the difficulties in emotion regulation scale. J ofPsychopathology Behav Assess. (2004) 26:41–5. doi: 10.1023/B:JOBA.0000007455.08539.94

[50] Wang L Liu H Du W Li Z. Test of difficulties in emotion regulation scale in chinese people. China J Health Psychol. (2007) 04):336–40. doi: 10.13342/j.cnki.cjhp.2007.04.020

[51] Zimet GD Powell SS Farley GK Werkman S Berkoff KA. Psychometric characteristics of the multidimensional scale of perceived social support. J Pers Assess. (1990) 55:610–7. doi: 10.1080/00223891.1990.9674095 2280326

[52] Zhao L Sun Q Guo Y Yan R Lv Y. Mediation effect of perceived social support and resilience between physical disability and depression in acute stroke patients in China: A cross-sectional survey. J Affect Disord. (2022) 308:155–9. doi: 10.1016/j.jad.2022.04.034 35429523

[53] Beck AT Steer RA Carbin MG. Psychometric properties of the beck depression inventory: twenty-five years of evaluation. Clin Psychol Rev. (1988) 8:77–100. doi: 10.1016/0272-7358(88)90050-5

[54] Li H Fu R Zou Y Cui Y. Predictive roles of three-dimensional psychological pain, psychache, and depression in suicidal ideation among Chinese college students. Front Psychol. (2017) 8:1550. doi: 10.3389/fpsyg.2017.01550 28955271 PMC5601061

[55] Podsakoff PM MacKenzie SB Lee J-Y Podsakoff NP. Common method biases in behavioral research: A critical review of the literature and recommended remedies. J Appl Psychol. (2003) 88:879–903. doi: 10.1037/0021-9010.88.5.879 14516251

[56] Dahlin M Joneborg N Runeson B. Stress and depression among medical students: A cross-sectional study. Med Educ. (2005) 39:594–604. doi: 10.1111/j.1365-2929.2005.02176.x 15910436

[57] Meng X Su H Li C. Effect of self-efficacy on bedtime procrastination among Chinese University students: A moderation and mediation model. Front Psychol. (2022) 13:863523. doi: 10.3389/fpsyg.2022.863523 35651571 PMC9149283

[58] Mostafa A Hoque R Mostafa M Rana M Mostafa F. Empathy in undergraduate medical students of Bangladesh: psychometric analysis and differences by gender, academic year, and specialty preferences. Int Scholarly Res Notices. (2014) 2014. doi: 10.1155/2014/375439 PMC4004052 25006522

[59] Qi Y Zhao M Geng T Tu Z Lu Q Li R. The relationship between family functioning and social media addiction among university students: A moderated mediation model of depressive symptoms and peer support. BMC Psychol. (2024) 12:341. doi: 10.1186/s40359-024-01818-2 38858753 PMC11165749

[60] Hayes AF ed. Process: A Versatile Computational Tool for Observed Variable Mediation, Moderation, and Conditional Process Modeling 1. White Paper (2012).

[61] Klumparendt A Nelson J Barenbrügge J. Associations between childhood maltreatment and adult depression: A mediation analysisassociations between childhood maltreatment and adult depression: A mediation analysis. BMC Psychiatry. (2019) 19:36. doi: 10.1186/s12888-019-2016-8 30669984 PMC6343339

[62] Cong E Li Y Shao C Chen J Wu W Shang X. Childhood sexual abuse and the risk for recurrent major depression in chinese women. psychol Med. (2012) 42:409–17. doi: 10.1017/S0033291711001462 PMC3250087 21835095

[63] Adams J Mrug S Knight DC. Characteristics of child physical and sexual abuse as predictors of psychopathology. Child Abuse Negl. (2018) 86:167–77. doi: 10.1016/j.chiabu.2018.09.019 PMC6289670 30308347

[64] Robert M Post MD. Transduction of psychosocial stress into the neurobiology of recurrent affective disorder. Am J Psychiatry. (1992) 149:999–1010. doi: 10.1176/ajp.149.8.999 1353322

[65] Tian L Zhou Z Huebner ES. Association between emotional abuse and depressive symptoms in Chinese children: the mediating role of emotion regulation. Child Abuse Negl. (2023) 139:106135. doi: 10.1016/j.chiabu.2023.106135 36924624

[66] Gruhn MA Compas BE. Effects of maltreatment on coping and emotion regulation in childhood and adolescence: A meta-analytic review. Child Abuse Negl. (2020) 103:104446. doi: 10.1016/j.chiabu.2020.104446 32200195 PMC12352122

[67] Thompson RA. Emotional regulation and emotional development. Educ Psychol Rev. (1991) 3:269–307. doi: 10.1007/BF01319934

[68] Cloitre M Stovall-McClough C Zorbas P Charuvastra A. Attachment organization, emotion regulation, and expectations of support in a clinical sample of women with childhood abuse histories. J Trauma Stress. (2008) 21:282–9. doi: 10.1002/jts.20339 18553408

[69] Crittenden PM. A dynamic-maturational model of attachment. Aust New Z J Family Ther. (2006) 27:105–15. doi: 10.1002/j.1467-8438.2006.tb00704.x

[70] Cooke JE Kochendorfer LB Stuart-Parrigon KL Koehn AJ Kerns KA. Parent-child attachment and children’s experience and regulation of emotion: A meta-analytic review. Emotion. (2019) 19:1103–26. doi: 10.1037/emo0000504 30234329

[71] Bernstein EE McNally RJ. Acute aerobic exercise helps overcome emotion regulation deficits. Cogn Emotion. (2016) 31:834–43. doi: 10.1080/02699931.2016.1168284 27043051

[72] Ishii K Shibata A Adachi M Oka K. Association of physical activity and sedentary behavior with psychological well-being among Japanese children. Perceptual Motor Skills. (2016) 123:445–59. doi: 10.1177/0031512516662645 27516410

[73] McLaughlin LE Luberto CM O’Bryan EM Kraemer KM McLeish AC. The indirect effect of positive affect in the relationship between trait mindfulness and emotion dysregulation. Pers Individ Dif. (2019) 145:70–4. doi: 10.1016/j.paid.2019.03.020 PMC8221243 34168391

[74] Cheong EV Sinnott C Dahly D Kearney PM. Adverse childhood experiences (Aces) and later-life depression: perceived social support as a potential protective factor. BMJ Open. (2017) 7:e013228. doi: 10.1136/bmjopen-2016-013228 PMC5588961 28864684

[75] Ochi S Dwivedi Y. Dissecting early life stress-induced adolescent depression through epigenomic approach. Mol Psychiatry. (2022) 28:141–53. doi: 10.1038/s41380-022-01907-x PMC9812796 36517640

[76] La Greca AM Lai BS Joormann J Auslander BB Short MA. Children’s risk and resilience following a natural disaster: genetic vulnerability, posttraumatic stress, and depression. J Affect Disord. (2013) 151:860–7. doi: 10.1016/j.jad.2013.07.024 24035489

[77] Allen L Dwivedi Y. Microrna mediators of early life stress vulnerability to depression and suicidal behavior. Mol Psychiatry. (2019) 25:308–20. doi: 10.1038/s41380-019-0597-8 PMC6974433 31740756

[78] Chen FS Kumsta R von Dawans B Monakhov M Ebstein RP Heinrichs M. Common oxytocin receptor gene (Oxtr) polymorphism and social support interact to reduce stress in humans. Proc Natl Acad Sci. (2011) 108:19937–42. doi: 10.1073/pnas.1113079108 PMC3250137 22123970

[79] Lee JY Stewart R Kang HJ Kim JW Jhon M Kim SW. Childhood abuse, social support, and long-term pharmacological treatment outcomes in patients with depressive disorders. Front Psychiatry. (2022) 13:803639. doi: 10.3389/fpsyt.2022.803639 35185652 PMC8847738

[80] Grave U Glanert S Borchfeld K Outzen J Schweiger U Fassbinder E. Differential effect of childhood emotional abuse on present social support in borderline disorder and depression: A cross-sectional study. Eur J Psychotraumatol. (2021) 12:1968612. doi: 10.1080/20008198.2021.1968612 34868477 PMC8635563

[81] Struck N Krug A Feldmann M Yuksel D Stein F Schmitt S. Attachment and social support mediate the association between childhood maltreatment and depressive symptoms. J Affect Disord. (2020) 273:310–7. doi: 10.1016/j.jad.2020.04.041 32421618

[82] Lee CYS Dik BJ. Associations among stress, gender, sources of social support, and health in emerging adults. Stress Health. (2016) 33:378–88. doi: 10.1002/smi.2722 27762485

[83] Urano Y Ikeda T. Perceived social support moderates the association between emotion regulation and psychological distress: A cross-sectional study among Japanese adults. Psychology Health Med. (2021) 26:1195–205. doi: 10.1080/13548506.2020.1802051 32804546

[84] Karatekin C Ahluwalia R. Effects of adverse childhood experiences, stress, and social support on the health of college students. J Interpersonal Violence. (2016) 35:150–72. doi: 10.1177/0886260516681880 27920360

[85] Wen LY Shi LX Zhu LJ Zhou MJ Hua L Jin YL. Associations between chinese college students’ Anxiety and depression: A chain mediation analysis. PloS One. (2022) 17:e0268773. doi: 10.1371/journal.pone.0268773 35653383 PMC9162318

[86] Hobfoll SE. Social support: the movie. J Soc Pers Relat. (2009) 26:93–101. doi: 10.1177/0265407509105524

[87] Park J Lee DS Shablack H Verduyn P Deldin P Ybarra O. When perceptions defy reality: the relationships between depression and actual and perceived facebook social support. J Affect Disord. (2016) 200:37–44. doi: 10.1016/j.jad.2016.01.048 27126138

[88] Zhao C Xu H Lai X Yang X Tu X Ding N. Effects of online social support and perceived social support on the relationship between perceived stress and problematic smartphone usage among Chinese undergraduates. Psychol Res Behav Manage. (2021) 14:529–39. doi: 10.2147/prbm.S302551 PMC8106527 33976576

