# Supplementary material for: The influence of emotion dysregulation and perceived social support on the link between childhood emotional abuse and depressive symptoms in college students: a moderated mediation model
Source: Front Psychiatry. 2025 Apr 22;16:1538390. doi: 10.3389/fpsyt.2025.1538390 (PMC12054248; doi:10.3389/fpsyt.2025.1538390)
Supplement: Supplementary file 1 [file DataSheet1.docx]

**Supplementary Materials**

**Results**

**1.Male group**

1.1 Demographics characteristics and preliminary statistics

The mean age of the male sample was 18.44 (SD = 0.77, age of 18-24) years. The male sample’s average EA score was 6.44 (SD = 2.50). 98 participants (14.98%, score of 10-25) reported having experienced emotional abuse during childhood. The mean BDI score for the male sample was 4.44 (SD = 6.26). 110 participants (16.82%) showed scores indicative of mild to severe depressive symptoms. Specifically, 82 participants (12.54%) had mild depressive symptoms (score of 10-18), 22 participants (4.17%) had moderate depressive symptoms (score of 19-29), and 6 participants (0.58%) had severe depressive symptoms (score of 30-63). **Table S1** presents the summary of the descriptive statistics for all variables and the percentage of depressive symptoms.

| **Table S1** Descriptive statistics of the studied variables in the male and female group, respectively. | | | | | |
| --- | --- | --- | --- | --- | --- |
|  | Variable | Mean | SD | Range | n/N (%) |
| Male (n=654) | EA | 6.44 | 2.50 | 5-21 |  |
|  | DERS | 79.21 | 22.11 | 36-146 |  |
|  | PSSS | 64.79 | 14.21 | 12-84 |  |
|  | BDI | 4.44 | 6.26 | 0-39 |  |
|  | Mild depression | 13.09 | 2.49 | 10-18 | 12.54% |
|  | Moderate depression | 21.91 | 2.58 | 19-29 | 4.17% |
|  | Severe depression | 34.33 | 2.86 | 30-63 | 0.58% |
| Female (n=1074) | EA | 6.76 | 2.67 | 5-25 |  |
|  | DERS | 81.77 | 22.45 | 36-146 |  |
|  | PSSS | 64.82 | 12.42 | 22-84 |  |
|  | BDI | 5.18 | 6.35 | 0-45 |  |
|  | Mild depression | 13.10 | 2.51 | 10-18 | 15.08% |
|  | Moderate depression | 22.78 | 2.76 | 19-29 | 4.66% |
|  | Severe depression | 33.75 | 6.65 | 30-63 | 0.37% |

SD, standard deviation. EA, total score of the Emotional Abuse subscale of the Childhood Trauma Questionnaire-Short Form; DERS, total score of the Difficulties in Emotion Regulation Scale. PSSS, total score of the Perceived Social Support Scale; BDI, depressive score using the Beck Depression Inventory; n/N (%), percentage of different levels of depression.

In addition, we use independent-samples t-tests to examine the gender effects on the studied variables, as shown in **Table S2**. The two groups of subjects did not differ with respect to PSSS. However, significant gender differences were observed in EA (*t* = -2.41, *p* < 0.016), DERS (*t* = -2.31, *p* < 0.021) and BDI (*t* = -2.36, *p* < 0.018). Female subjects exhibited higher levels of EA, DERS, and BDI scores compared to male subjects.

| **Table S2** Gender effects on the studied variables**.** | | | | | | | |
| --- | --- | --- | --- | --- | --- | --- | --- |
|  | **Male (n=654)** | |  | **Female (n=1074)** | | ***t*-value** | ***p*-value** |
|  | Mean | SD |  | Mean | SD |  |  |
| **EA** | 6.44 | 2.50 |  | 6.76 | 2.67 | -2.41 | 0.016 |
| **DERS** | 79.21 | 22.11 |  | 81.77 | 22.45 | -2.31 | 0.021 |
| **PSSS** | 64.79 | 14.21 |  | 64.82 | 12.42 | -0.05 | 0.961 |
| **BDI** | 4.44 | 6.26 |  | 5.18 | 6.35 | -2.36 | 0.018 |
| Independent sample t-tests was used to examine gender differences. SD, standard deviation. EA, total score of the Emotional Abuse subscale of the Childhood Trauma Questionnaire-Short Form; DERS, total score of the Difficulties in Emotion Regulation Scale. PSSS, total score of the Perceived Social Support Scale; BDI, depressive score using the Beck Depression Inventory. | | | | | | | |

1.2 The correlation among childhood emotional abuse, perceived social support, emotion dysregulation and depression.

The results of the bivariate correlation analyses in the male group are delineated in **Table S3**. As expected, both EA (*r* = 0.437, *p* < 0.001) and DERS (*r* = 0.494, *p* < 0.001) were positively correlated with BDI. In contrast, PSSS (*r* = -0.372, *p* < 0.001) was negatively correlated with BDI. Furthermore, EA demonstrated a significant positive correlation with DERS (*r* = 0. 358, *p* < 0.001) and negative correlation with PSSS (*r* = -0.368, *p* < 0.001). Additionally, DERS was negatively correlated with PSSS (*r* = -0.412, *p* < 0.001). These results were consistent with the main results derived from all samples.

| **Table S3** Intercorrelations between the studied variables in the male and female group, respectively. | | | | |  |
| --- | --- | --- | --- | --- | --- |
|  | Variable | 1 | 2 | 3 | |
| Male (n=654) | 1. EA |  |  |  | |
|  | 2. DERS | 0.358^***^ |  |  | |
|  | 3. PSSS | -0.368^***^ | -0.412^***^ |  | |
|  | 4. BDI | 0.437^***^ | 0.494^***^ | -0.372^***^ | |
| Female (n=1074) | 1.EA |  |  |  | |
|  | 2.DERS | 0.381^***^ |  |  | |
|  | 3.PSSS | -0.315^***^ | -0.496^***^ |  | |
|  | 4.BDI | 0.452^***^ | 0.555^***^ | -0.365^***^ | |
| EA, total score of the Emotional Abuse subscale of the Childhood Trauma Questionnaire-Short Form; DERS, total score of the Difficulties in Emotion Regulation Scale; PSSS: total score of the Perceived Social Support Scale; BDI, depressive score using the Beck Depression Inventory. ^***^ *p*＜0.001. | | | | |  |

1.3 The mediating role of emotion dysregulation

The mediating effect of the DERS on the relationship between EA and BDI in the male group was detailed in **Table S4**. In Model 1, EA was positively associated with DERS (*B* = 0.363, *p* < 0.001). In Model 2, EA was positively associated with BDI (*B* = 0.436, *p* < 0.001). In Model 3, after controlling for EA, DERS was positively associated with BDI (*B* = 0.389, *p* < 0.001) in Model 3. Despite EA retaining a positive association with BDI (*B* = 0.295, *p* < 0.001) in Model 3, its impact on BDI was attenuated after controlling for DERS relative to Model 2.

In addition, we employed Hayes’ (2012) SPSS Process Macro, Model 4, to carry out a bootstrap analysis to reiterate the indirect effect of DERS in the association between EA and BDI, while controlling for age. The indirect effect of EA on BDI via the DERS (95% CI = [0.102, 0.187]) was significant. This result indicated that emotion dysregulation mediated the pathway from childhood emotional abuse to depression among Chinese college male students.

**Table S4** Results of the path analyses in the male group (n=654).

| Predictor variable | **DERS** | **BDI** | | |
| --- | --- | --- | --- | --- |
|  | Model 1 | Model 2 | Model 3 | Model 4 |
| Intercept | 0.920 | -0.184 | -0.542 | -0.284 |
| **Control variables** |  |  |  |  |
| Age (years old) | -0.050 | 0.010 | 0.029 | 0.012 |
| **Independent variable** |  |  |  |  |
| EA | 0.363^***^ | 0.436^***^ | 0.295^***^ | 0.171^***^ |
| **Mediator** |  |  |  |  |
| DERS |  |  | 0.389^***^ | 0.370^***^ |
| **Moderator** |  |  |  |  |
| PSSS |  | - |  | -0.127^***^ |
| **Interaction term** |  |  |  |  |
| EA × PSSS |  |  |  | -0.102^***^ |
| DERS × PSSS |  |  |  | -0.086^**^ |
| *R²* | 0.129 | 0.191 | 0.323 | 0.358 |
| *F* | 48.376^***^ | 76.879^***^ | 103.131^***^ | 60.126^***^ |
| Unstandardized regression coefficients are reported. EA, total score of the Emotional Abuse subscale of the Childhood Trauma Questionnaire-Short Form; DERS, total score of the Difficulties in Emotion Regulation Scale; PSSS, total score of the Perceived Social Support Scale; BDI, depressive score using the Beck Depression Inventory; ^**^ *p* < 0.01, ^***^ *p* < 0.001. | | | | |

1.4 The moderating effect of perceived social support

Our finding indicated that PSSS significantly moderated the direct influence of EA on BDI (*B* = -0.102, *p* < 0.001), and the link between DERS and BDI (*B* = -0.086, *p* < 0.01), as depicted in **Table S4**-Model 4. As presented in **Table S5**, the positive association between EA and BDI was no longer significant in males with high PSSS (95% CI = [-0.055, 0.192]). The simple slope analyses were used to show the significant interaction at one SD below and one SD above the average of PSSS (See **Figure S1**). Specifically, the direct path between EA and BDI was significant in males with low PSSS (*B* = 0.273, *p* < 0.001), but was not significant in males with high PSSS (*B* = 0.069, *p* = 0.275). For the indirect path, the impact of DERS on BDI was significant in males with both low PSSS (*B* = 0.455, *p* < 0.001) and high PSSS (*B* = 0.284, *p* < 0.001). But the relationship between DERS and BDI is stronger in males with low PSSS and weaker in males with high PSSS (**Table S6**).

In addition, we also used Hayes’ (2012) SPSS Process Macro, Model 15 to carry out a bootstrap analysis to reiterate the moderated mediation effect, taking age as covariates. Our findings revealed that PSSS significantly moderated both the direct relationship between EA and BDI (*B* = 0.171, 95% CI = [0.089, 0.253]) and the indirect path between EA and BDI through DERS (*B*= 0.370, 95% CI = [0.299, 0.441]). The conditional direct and indirect effects between EA and BDI under different levels of PSSS were presented in **Table S5**. The pairwise contrasts of the conditional indirect effects were presented in **Table S6**. Pairwise contrast in the conditional indirect effects between EA and BDI under different levels of PSSS indicated that the indirect effect for males with high PSSS (indirect effect = 0.103, 95%CI = [0.067, 0.145]) was significantly weaker than for those with low PSSS (indirect effect = 0.165, 95%CI = [0.112, 0.229]), as the zero was excluded from the 95% CI. This may suggest that the connection between EA and BDI, mediated by DERS, is attenuated among males with higher PSSS.


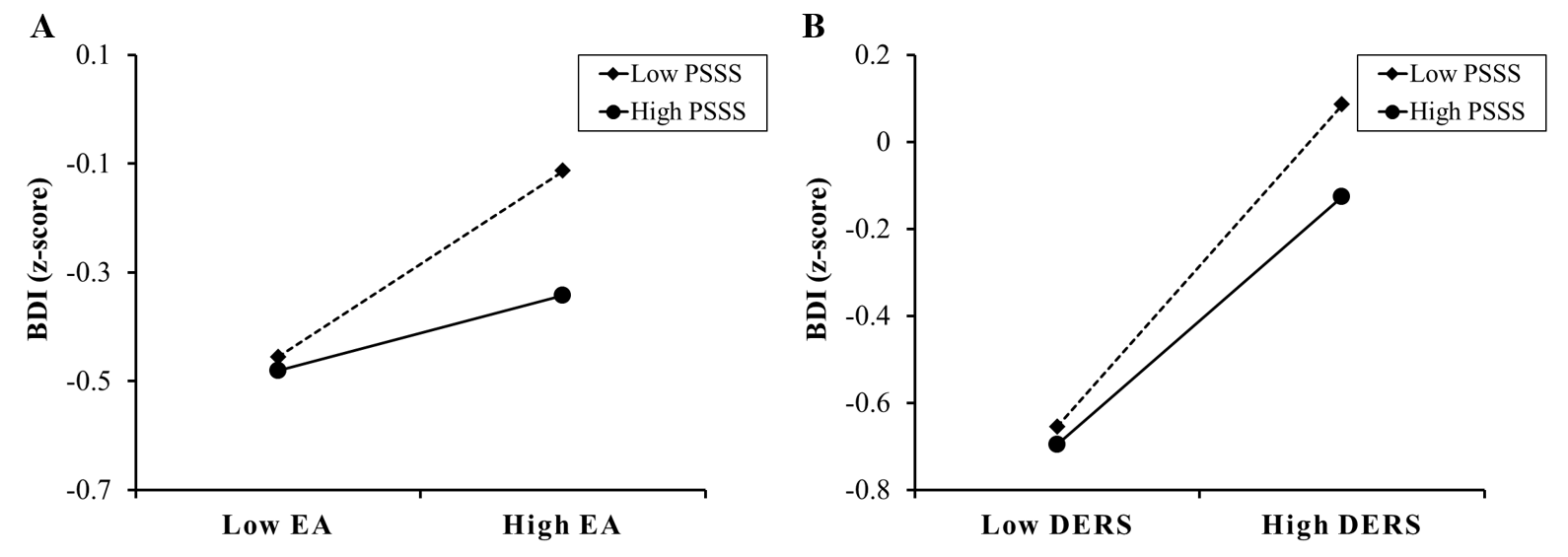


**Figure S1** The results of simple slope analyses in the male group. (A) Perceived social support moderates the effect of childhood emotional abuse on depressive scores. (B) Perceived social support moderates the effect of difficulties in emotional regulation on depressive scores. EA, total score of the Emotional Abuse subscale of the Childhood Trauma Questionnaire-Short Form; DERS, total score of the Difficulties in Emotion Regulation Scale; PSSS, total score of the Perceived Social Support Scale; BDI, depressive score using the Beck Depression Inventory.

| **Table S5** Conditional direct and indirect effects of EA on BDI under different levels of PSSS in the male group. | | | | | | |
| --- | --- | --- | --- | --- | --- | --- |
|  | **EA→BDI** | | | **EA→DERS→BDI** | | |
|  | Effect | BootLLCL | BootULCI | Effect | BootLLCL | BootULCI |
| Effect 1 (M - 1SD) | 0.273 | 0.201 | 0.345 | 0.165 | 0.112 | 0.229 |
| Effect 2 (M) | 0.171 | 0.089 | 0.253 | 0.134 | 0.096 | 0.179 |
| Effect 3 (M +1SD) | 0.069 | -0.055 | 0.192 | 0.103 | 0.067 | 0.145 |
| EA, total score of the Emotional Abuse subscale of the Childhood Trauma Questionnaire-Short Form; DERS, total score of the Difficulties in Emotion Regulation Scale; PSSS, total score of the Perceived Social Support Scale; BDI, depressive score using the Beck Depression Inventory; M, mean; SD, standard deviation; BootLLCI, lower limit of 95% confidence interval; BootULCI, upper limit of 95% confidence interval. | | | | | | |

| **Table S6** Pairwise contrasts between conditional indirect effects in the male and female group, respectively. | | | | | | |  |
| --- | --- | --- | --- | --- | --- | --- | --- |
|  |  | PSSS | Effect | BootSE | BootLLCL | BootULCI | |
| Male (n=654) | Pairwise contrasts between conditional indirect effects | Effect 2 - Effect 1 | -0.031 | 0.014 | -0.060 | -0.004 | |
|  |  | Effect 3 - Effect 1 | -0.060 | 0.029 | -0.120 | -0.007 | |
|  |  | Effect 3 - Effect 2 | -0.031 | 0.014 | -0.060 | -0.004 | |
| Female (n=1074) | Pairwise contrasts between conditional indirect effects | Effect 2 - Effect 1 | -0.033 | 0.013 | -0.060 | -0.011 | |
|  |  | Effect 3 - Effect 1 | -0.066 | 0.025 | -0.121 | -0.022 | |
|  |  | Effect 3 - Effect 2 | -0.033 | 0.013 | -0.060 | -0.011 | |

PSSS, total score of the Perceived Social Support Scale; BootSE, standard error of bootstrap; BootLLCI, lower limit of 95% confidence interval; BootULCI, upper limit of 95% confidence interval.

**2.Female group**

2.1 Demographics characteristics and preliminary statistics

The mean age of the female sample was 18.28 (SD = 0.59, age of 18-22) years. The female sample’s average EA score was 6.76 (SD = 2.67). 187 participants (17.40%, score of 9-25) reported having experienced emotional abuse during childhood. The mean BDI score for the female sample was 5.18 (SD = 6.35). 216 participants (20.11%) showed scores indicative of mild to severe depressive symptoms. Specifically,162 participants (15.08%) had mild depressive symptoms (score of 10-18), 50 participants (4.66%) had moderate depressive symptoms (score of 19-29), and 4 participants (0.37%) had severe depressive symptoms (score of 30-63). **Table S1** presents the summary of the descriptive statistics for all variables and the percentage of depressive symptoms.

2.2 The correlation among childhood emotional abuse, perceived social support, emotion dysregulation and depression.

The results of the bivariate correlation analyses in the female group are delineated in **Table S3**. As expected, both EA (*r* = 0.452, *p* < 0.001) and DERS (*r* = 0.555, *p* < 0.001) were positively correlated with BDI. In contrast, PSSS (*r* = -0.365, *p* < 0.001) was negatively correlated with BDI. Furthermore, EA demonstrated a significant positive correlation with DERS (*r* = 0. 381, *p* < 0.001) and negative correlation with PSSS (*r* = -0.315, *p* < 0.001). Additionally, DERS was negatively correlated with PSSS (*r* = -0.496, *p* < 0.001). These results were consistent with the main results derived from all samples.

2.3 The mediating role of emotion dysregulation

The mediating effect of the DERS on the relationship between EA and BDI in the female group was detailed in **Table S7**. In Model 1, EA was positively associated with DERS (*B* = 0.380, *p* < 0.001). In Model 2, EA was positively associated with BDI (*B* = 0.451, *p* < 0.001). In Model 3, after controlling for EA, DERS was positively associated with BDI (*B* = 0.447, *p* < 0.001). Despite EA retaining a positive association with BDI (*B* = 0.281, *p* <0.001) in Model 3, its impact on BDI was attenuated after controlling for DERS relative to Model 2.

In addition, we employed Hayes’ (2012) SPSS Process Macro, Model 4, to carry out a bootstrap analysis to reiterate the indirect effect of DERS in the association between EA and BDI, while controlling for age. The indirect effect of EA on BDI via the DERS (95% CI = [0.133, 0.214]) was significant. This result indicated that emotion dysregulation mediated the pathway from childhood emotional abuse to depression among Chinese college female students.

**Table S7** Results of the path analyses in the female group (n=1074).

| Predictor variable | **DERS** | **BDI** | | |
| --- | --- | --- | --- | --- |
|  | Model 1 | Model 2 | Model 3 | Model 4 |
| Intercept | -0.844 | -1.435 | -1.059 | -1.265 |
| **Control variables** |  |  |  |  |
| Age (years old) | 0.046 | 0.076 | 0.058 | 0.066 |
| **Independent variable** |  |  |  |  |
| EA | 0.380^***^ | 0.451^***^ | 0.281^***^ | 0.231^***^ |
| **Mediator** |  |  |  |  |
| DERS |  |  | 0.447^***^ | 0.428^***^ |
| **Moderator** |  |  |  |  |
| PSSS |  | - |  | -0.066^*^ |
| **Interaction term** |  |  |  |  |
| EA × PSSS |  |  |  | -0.045 |
| DERS × PSSS |  |  |  | -0.086^***^ |
| *R²* | 0.146 | 0.207 | 0.377 | 0.393 |
| *F* | 91.517^***^ | 139.460^***^ | 215.814^***^ | 138.296^***^ |
| Unstandardized regression coefficients are reported. EA, total score of the Emotional Abuse subscale of the Childhood Trauma Questionnaire-Short Form; DERS, total score of the Difficulties in Emotion Regulation Scale; PSSS, total score of the Perceived Social Support Scale; BDI, depressive score using the Beck Depression Inventory; ^*^ *p* < 0.05, ^***^ *p* < 0.001. | | | | |

2.4 The moderating effect of perceived social support

Our finding indicated that PSSS significantly moderated the link between DERS and BDI (*B* = -0.086, *p* < 0.001), as depicted in **Table S7**-Model 4. In addition, simple slope analyses were used to show the significant interaction at one SD below and one SD above the average of PSSS (See **Figure S2**). This result indicated that regardless of the extent of PSSS, heightened DERS is linked to greater depressive symptoms. Specifically, the indirect path between DERS and BDI was significant in females in low PSSS (*B* = 0.514, *p* < 0.001) and high PSSS (*B* = 0.341, *p* < 0.001). But the relationship between DERS and BDI is stronger in females with low PSSS and weaker in males with high PSSS (**Table S6**).

In addition, we also used Hayes’ (2012) SPSS Process Macro, Model 14 to carry out a bootstrap analysis to reiterate the moderated mediation effect, taking age as covariates. Our findings revealed that PSSS significantly moderated the indirect path between EA and BDI through DERS (*B* = 0.428, 95% CI = [0.371, 0.484]). The conditional direct and indirect effects between EA and BDI under different levels of PSSS were presented in **Table S8**. The pairwise contrasts of the conditional indirect effects between EA and BDI were presented in **Table S6**. Pairwise contrast in the conditional indirect effects between EA and BDI under different levels of PSSS indicated that the indirect effect for female with high PSSS (indirect effect = 0.130, 95%CI = [0.097, 0.170]) was significantly weaker than for those with low PSSS (indirect effect = 0.195, 95%CI = [0.145, 0.260), as the zero was excluded from the 95% CI. This result may suggest that among individuals with greater PSSS, the connection between EA and BDI, mediated by DERS, is attenuated.


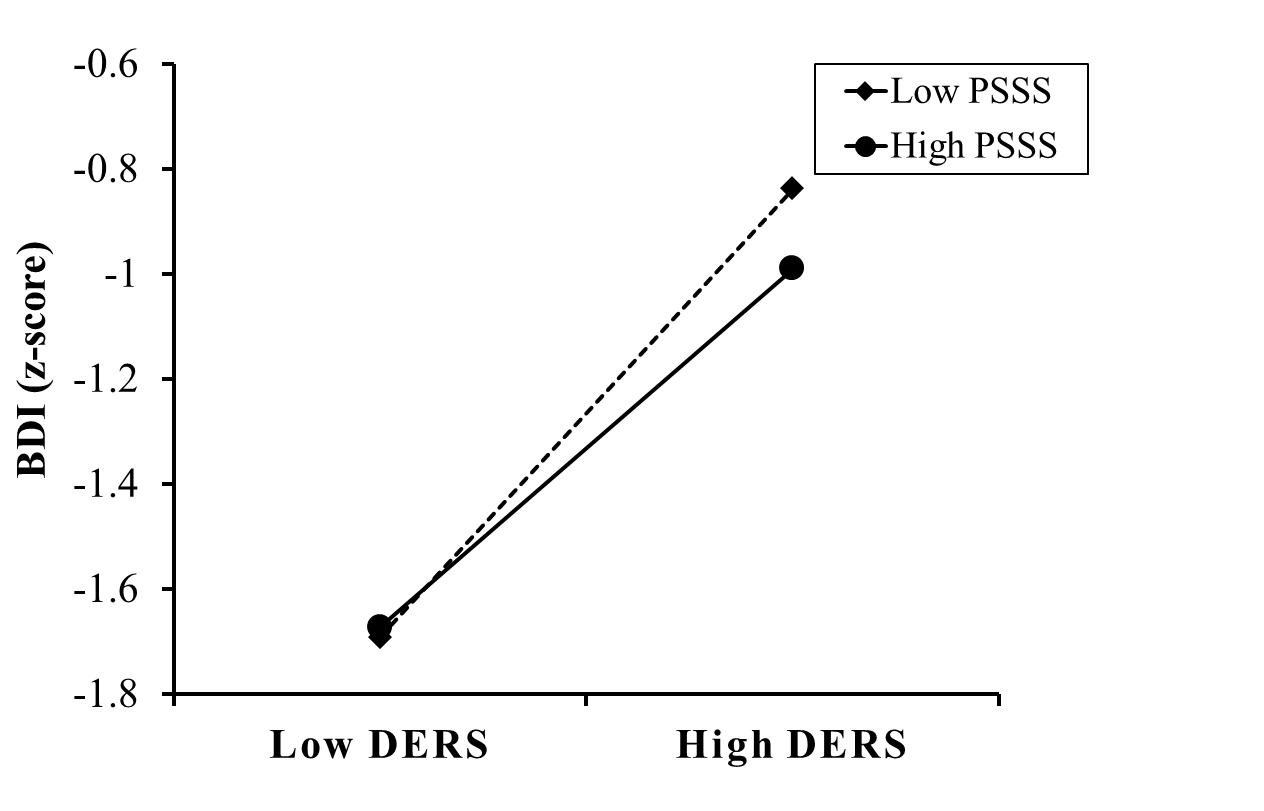


**Figure S2** Perceived social support moderates the effect of difficulties in emotional regulation on depressive scores in the female group. DERS, total score of the Difficulties in Emotion Regulation Scale; PSSS, total score of the Perceived Social Support Scale; BDI, depressive score using the Beck Depression Inventory.

| **Table S8** Conditional indirect effects of EA on BDI under different levels of PSSS in the female group. | | | | | | | |  |
| --- | --- | --- | --- | --- | --- | --- | --- | --- |
|  | **EA→DERS→BDI** | | | | | |  |  |
|  | Effect | | BootLLCL | | BootULCI | |  |  |
| Effect 1 (M - 1SD) | | 0.195 | | 0.145 | | 0.260 | |  |
| Effect 2 (M) | | 0.163 | | 0.126 | | 0.208 | |  |
| Effect 3 (M +1SD) | | 0.130 | | 0.097 | | 0.170 | |  |
| EA, total score of the Emotional Abuse subscale of the Childhood Trauma Questionnaire-Short Form; DERS, total score of the Difficulties in Emotion Regulation Scale; PSSS, total score of the Perceived Social Support Scale; BDI, depressive score using the Beck Depression Inventory; M, mean; SD, standard deviation; BootLLCI, lower limit of 95% confidence interval; BootULCI, upper limit of 95% confidence interval. | | | | | | | | |
